# Eicosapentaenoic acid prevents arterial calcification in *klotho* mutant mice

**DOI:** 10.1371/journal.pone.0181009

**Published:** 2017-08-03

**Authors:** Kazufumi Nakamura, Daiji Miura, Yukihiro Saito, Kei Yunoki, Yasushi Koyama, Minoru Satoh, Megumi Kondo, Kazuhiro Osawa, Omer F. Hatipoglu, Toru Miyoshi, Masashi Yoshida, Hiroshi Morita, Hiroshi Ito

**Affiliations:** 1 Department of Cardiovascular Medicine, Okayama University Graduate School of Medicine, Dentistry and Pharmaceutical Sciences, Okayama, Japan; 2 Department of Basic Medicine, Nagano College of Nursing, Komagane, Japan; 3 Department of Cardiology, Sakurabashi-Watanabe Hospital, Osaka, Japan; 4 Department of Nephrology and Hypertension, Kawasaki Medical School, Kurashiki, Japan; 5 Department of Cardiovascular Therapeutics, Okayama University Graduate School of Medicine, Dentistry, and Pharmaceutical Sciences, Okayama, Japan; Albany Medical College, UNITED STATES

## Abstract

**Background:**

The *klotho* gene was identified as an “aging-suppressor” gene that accelerates arterial calcification when disrupted. Serum and vascular klotho levels are reduced in patients with chronic kidney disease, and the reduced levels are associated with arterial calcification. Intake of eicosapentaenoic acid (EPA), an n-3 fatty acid, reduces the risk of fatal coronary artery disease. However, the effects of EPA on arterial calcification have not been fully elucidated. The aim of this study was to determine the effect of EPA on arterial calcification in *klotho* mutant mice.

**Methods and results:**

Four-week-old *klotho* mutant mice and wild-type (WT) mice were given a diet containing 5% EPA (EPA food, *klotho* and WT: n = 12, each) or not containing EPA (control food, *klotho* and WT: n = 12, each) for 4 weeks. Calcium volume scores of thoracic and abdominal aortas assessed by computed tomography were significantly elevated in *klotho* mice after 4 weeks of control food, but they were not elevated in *klotho* mice after EPA food or in WT mice. Serum levels of EPA and resolvin E1, an active metabolite of EPA, in EPA food-fed mice were significantly increased compared to those in control food-fed mice. An oxidative stress PCR array followed by quantitative PCR revealed that NADPH oxidase-4 (*NOX4*), an enzyme that generates superoxide, gene expression was up-regulated in arterial smooth muscle cells (SMCs) of *klotho* mice. Activity of NOX was also significantly higher in SMCs of *klotho* mice than in those of WT mice. EPA decreased expression levels of the NOX4 gene and NOX activity. GPR120, a receptor of n-3 fatty acids, gene knockdown by siRNA canceled effects of EPA on NOX4 gene expression and NOX activity in arterial SMCs of *klotho* mice.

**Conclusions:**

EPA prevents arterial calcification together with reduction of NOX gene expression and activity via GPR120 in *klotho* mutant mice.

## Introduction

Vascular calcification increases with aging and is highly prevalent in patients with atherosclerosis, diabetes mellitus and chronic kidney disease (CKD) [1]. Coronary artery calcium assessed by computed tomography (CT) provides independent incremental information in addition to traditional risk factors for the prediction of coronary heart disease and all-cause mortality [2, 3].

The *klotho* gene was identified as an “aging-suppressor” gene in mice, and it was shown that disruption of the gene results in acceleration of arterial calcification [4]. We and other investigators have reported that expression levels of serum and local vascular klotho are reduced in patients with CKD and that the decrease in expression level of klotho is associated with arterial calcification and stiffness in patients with CKD [5–7].

Intake of eicosapentaenoic acid (EPA), an n-3 fatty acid, reduces the risk of fatal coronary artery disease [8]. Several studies have revealed that EPA prevents vascular calcification. EPA attenuates arterial medial calcification in warfarin-induced rat models.[9] EPA prevents vascular calcification by inhibiting palmitic acid-induced mineralization of human arterial smooth muscle cells (SMCs) [10]. However, the effects of EPA on arterial calcification such as an association with klotho have not been fully elucidated.

The aim of this study was to determine the effect of EPA on arterial calcification assessed by CT in *klotho* mutant (*kl/kl*) mice. Furthermore, since oxidative stress is associated with the development of vascular calcification [11, 12], we assessed the effects of EPA on gene expression related to oxidative stress in SMCs of *kl/kl* mice.

## Materials and methods

### Animals

Klotho homozygous mutant (*kl/kl*) mice were purchased from CLEA Japan. Four-week-old klotho mutant (*kl/kl*) mice (n = 24, 12 males & 12 females) and wild-type (WT) mice (n = 24, 12 males & 12 females) were given a diet containing 5% EPA (Mochida Pharmaceutical Co. Ltd) (EPA food, klotho and WT: n = 12, each) or not containing EPA (control food, klotho and WT: n = 12, each) for 4 weeks. All animal protocols were approved and conducted according to the recommendations of Okayama University on Animal Care and Use. The animal procedures performed conform to the NIH guidelines (Guide for the Care and Use of Laboratory Animals).

### CT Image acquisition and aortic calcification volume quantification

The mice were anesthetized with inhalation of isoflurane. Image acquisitions were performed using multi-detector CT (FX3000 Pre-Clinical Imaging System, TriFoil Imaging Inc.) before and after 4 weeks of feeding. All images were acquired during an inspiratory breath hold, with tube voltage of 120 kV and single-slice thickness of 192 μm.

Calcium volume score of the thoracic and abdominal aorta was calculated by multiplying the number of voxels (Vn) with the voxel volume (Vv)[13] using the volume-rendering method by extracting the area ≥400 Hounsfield units within the entire aorta.

### Serum levels of EPA, arachidonic acid, inorganic phosphorus, calcium and resolvin E1

Serum levels of EPA and arachidonic acid (AA) were measured by gas chromatographic assay (SRL Inc. Tokyo). Serum inorganic phosphorus (Pi) levels were measured by method using molybdate (SRL Inc. Tokyo). Serum levels of calcium (Ca) were determined by method using arsenazo III (SRL Inc. Tokyo). Serum mouse resolvin E1 levels were measured using a commercially available enzyme-linked immunosorbent assay kit (MyBioSource Inc., San Diego, USA).

### Culture of arterial SMCs

After CT image acquisitions, all animals were anaesthetized and euthanized with an intraperitoneal injection of pentobarbital (50 mg/kg). Then the thoracic and abdominal aortas were removed from *kl/kl* mice and WT mice. SMCs were isolated from the thoracic and abdominal aortas by the explant culture method as described previously [14–16]. Thoracic and abdominal aortas were disaggregated with collagenase and cut into 2-mm-long sections, and then the adventitia layer was removed. Vessels were plated on a 6-well plate with Dulbecco’s modified Eagle’s medium (DMEM; Gibco, Grand Island, NY, USA) supplemented with 10% fetal bovine serum (FBS; Sigma) and 0.1 mg/mL kanamycin (Sigma) and incubated in a humidified 5% CO_2_ atmosphere at 37°C. The culture medium was changed every 3 days. After reaching confluence, the cells were subcultured by treatment with trypsin (0.05%)/ethylenediaminetetraacetic acid (EDTA) (0.02%). Cells between passages 3 to 5 were used for all experiments.

### Mouse oxidative stress PCR array and quantitative PCR

Oxidative stress-focused gene expression profiling of SMCs of a *kl/kl* mouse and a WT mouse was performed with the RT^2^ Profiler PCR Array System using the mouse oxidative stress PCR array (SABiosciences, a QIAGEN company) according to the manufacturer’s instructions. The array measures 84 key genes involved in oxidative stress. Total RNA from arterial SMCs was extracted using RNeasy Mini Kit (QIAGEN). Complementary DNA was synthesized from 1 μg of total RNA using ReverTra Ace (Toyobo Life Science, Tokyo) as prescribed in the manual and subjected to PCR amplification. Expression of mRNA was measured by reverse transcription PCR (RT-PCR) using an ABI PRISM 7300 sequence detector system (Applied Biosystems).

For quantitative PCR, arterial SMCs were reseeded in a 10-cm culture dish at a density of 5 x 10^4^ cells/well. After 24 hours, arterial SMCs were treated with EPA (20 μmol/L) (Sigma) dissolved in DMSO (Sigma) or 0.08% DMSO as a control. After 24 hours of incubation, total RNA was extracted from the SMCs and complementary DNA was synthesized as described above. Quantitative RT-PCR was performed with primers for cytoglobin (*Cygb*) (PPM28233A, SABiosciences, a QIAGEN company), glutathione peroxidase 3 (*GPX3*) (PPM06171A, SABiosciences, a QIAGEN company) or *GAPDH* (PPM02946E, SABiosciences, a QIAGEN company) in combination with RT^2^ SYBR Green qPCR Master Mix (SABiosciences, a QIAGEN company). Expression of mRNA was measured by RT-PCR using an ABI PRISM 7300 sequence detector system (Applied Biosystems). The quantitative PCR data were processed by a standard curve method. Expression levels were normalized against *GAPDH*.

### RT-PCR and quantitative PCR of NAD(P)H Oxidase 4 (NOX4) gene

Arterial SMCs were reseeded in 10-cm culture dish at a density of 5 x 10^4^ cells/well. After 24 hours, arterial SMCs were treated with EPA (20 μmol/L) (Sigma) dissolved in DMSO (Sigma) or 0.08% DMSO as a control. After 24 hours of incubation, total RNA was isolated from the SMCs using TRIzol (Life Technologies Japan). Reverse transcriptase reactions were performed using a Ready-To-Go T-Primed First-Strand Kit (GE Healthcare Japan, Tokyo, Japan) for first-strand cDNA synthesis. Real-time quantitative PCR was performed using the ABI Prism 7700 sequence detection system (Life Technologies Japan). Data were expressed as copy number relative to that of 18 S rRNA. The primers and probe used for TaqMan analysis of mouse Nox4 were described in our previous report [17]. TaqMan probes consist of the fluorophore 6-carboxyfluorescein (FAM) covalently attached to the 5’ end of the oligonucleotide probe and the quencher tetramethylrhodamine (TAMRA) at the 3’ end. In detail, the primers and probe for mouse NOX4 were as follows: 5’-cctttgcctccattctcaag-3’ (forward primer), 5’-caggtctgcaaaccactcaa-3’ (reverse primer) and 5′-FAM-ctggctgtgcagggacacgc-TAMRA-3’ (TaqMan probe).

### Lucigenin chemiluminescence assay of NAD(P)H Oxidase (NOX) activity

Arterial SMCs were prepared in the same manner as that described for quantitative PCR of NOX4. NOX activity of arterial SMCs was measured using lucigenin chemiluminescence (units/min/mg) as described previously [18]. Briefly, proteins from 5 × 10^4^ SMCs were diluted in modified Hepes buffer (140 mmol/L NaCl, 5 mmol/L KCl, 0.8 mmol/L MgCl_2_, 1.8 mmol/L CaCl_2_, 1 mmol/L Na_2_HPO_4_, 25 mmol/L Hepes, and 1% glucose, pH 7.2) and distributed (100 mg per well) onto a 96-well microplate. NADPH (100 μmol/L) and dark-adapted lucigenin (5 μmol/L; Sigma-Aldrich Japan, Tokyo, Japan) were added just before reading. Lucigenin chemiluminescence was recorded for 5 min and was stopped by the addition of 50 mM Tiron (Sigma-Aldrich Japan, Tokyo, Japan) to observe how the chemiluminescence was detected as superoxide. Lucegenin chemiluminescence was expressed as units per minute per milligram of protein (unit/min/mg). The data are shown as relative chemiluminescence intensity to WT Control. Experiments were performed in triplicate.

### Expression of G-protein-coupled receptor 120 (GPR120) mRNA

Total RNA was isolated from cultured SMCs using RNeasy Mini Kit (QIAGEN) as previously described [19]. First-strand cDNA was synthesized using ReverTra Ace (Toyobo Life Science, Tokyo). The primers for GPR120 were 5’-ccataaatctagtgctcgct-3’ (forward primer) and 5’-tgcggaagagtcggtagtct-3’ (reverse primer) as previously described [20].

### GPR120 knockdown by siRNA

To knock down GPR120, 5 μmol/L small interfering RNA (siRAN, s200889, Ambion) was transfected into mouse vascular smooth muscle cells explanted from the aorta using Lipofectamine RNAiMAX (Invitrogen) according to the manufacturer’s instructions. We confirmed Gpr120 downregulation by the PCR method. Total RNA was extracted using Trizol (Invitrogen) and Purelink RNA Mini Kit (Invitrogen). Complementary DNA was synthesized from 1 μg total RNA using Superscript III with Oligo(dT) primers (Invitrogen) according to the manufacturer’s instructions and subjected to PCR amplification. Taq DNA polymerase (Roche Applied Science) was used for RT-PCR. PCR products were subjected to electrophoresis in 2% agarose gels and stained with ethidium bromide. Primer pairs were as follows: GPR120 forward, tgcccctctgcatcttgttc; GPR120 reverse, cgcgatgctttcgtgatctg; GAPDH forward, catggccttccgtgttccta; and GAPDH reverse, tgcctgcttcaccaccttct. PCR product sizes were 202 bp and 106 bp, respectively, and the annealing temperature was 60°C.

### Statistical analysis

Data are expressed as mean ± standard error (SE). Data that were not normally distributed are expressed as median and interquartile range (IQR: 25%-75%). Statistical analysis was performed by Student’s *t* test or the chi-squared test for paired data or one-way ANOVA with comparison of different groups by Dunnett’s *post hoc* test. Values of P < 0.05 were considered to be significant.

## Results

### Changes in calcium volume score

**Fig 1:**
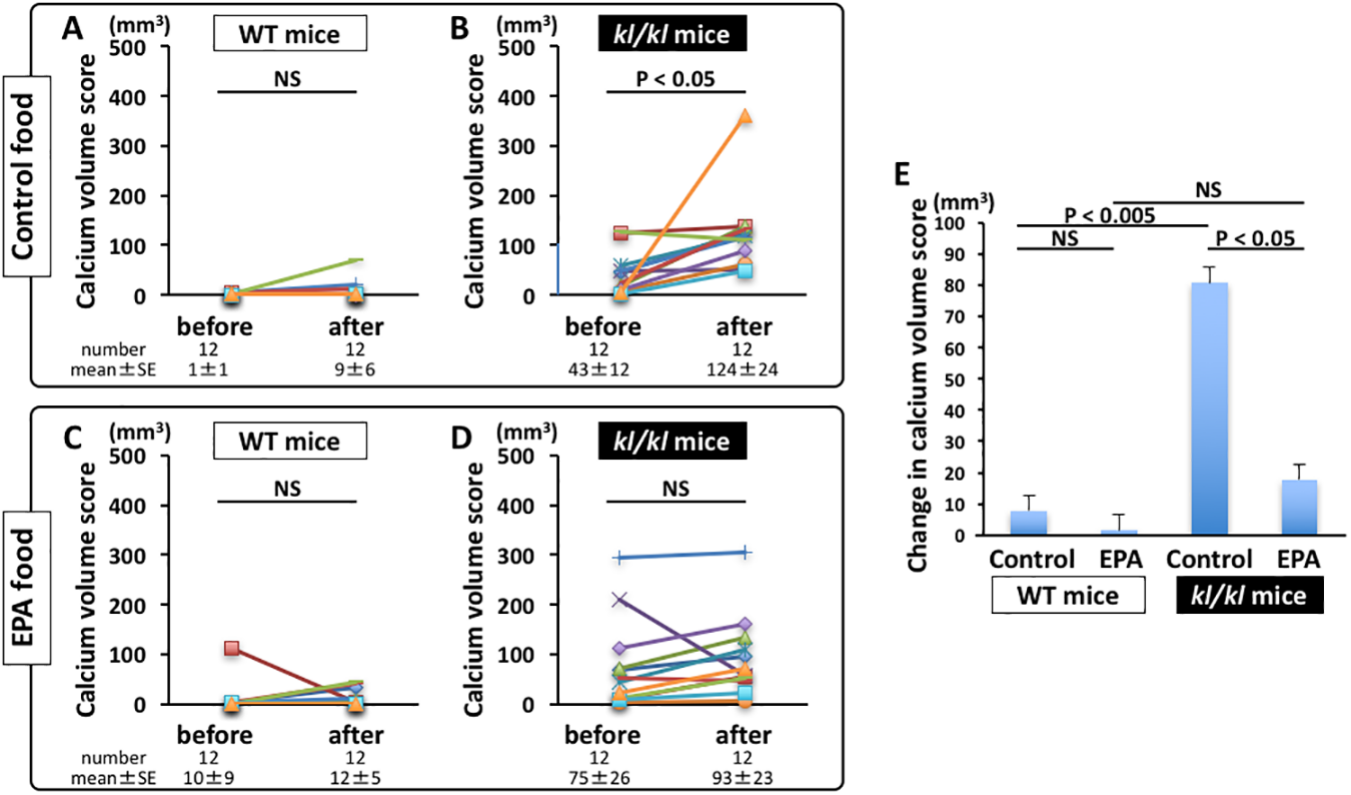
Changes in calcium volume score. A and B, Calcium volume scores in wild-type (WT) mice (A) and *klotho* mutant (*kl/kl)* mice (B) before and after 4 weeks of control food (n = 12, each). C and D, Calcium volume scores in WT mice (C) and *kl/kl* mice (D) before and after 4 weeks of EPA food (n = 12, each). E, Changes in calcium volume score in WT mice and *kl/kl* mice before and after 4 weeks of control and EPA food (n = 12, each).

**Fig 2:**
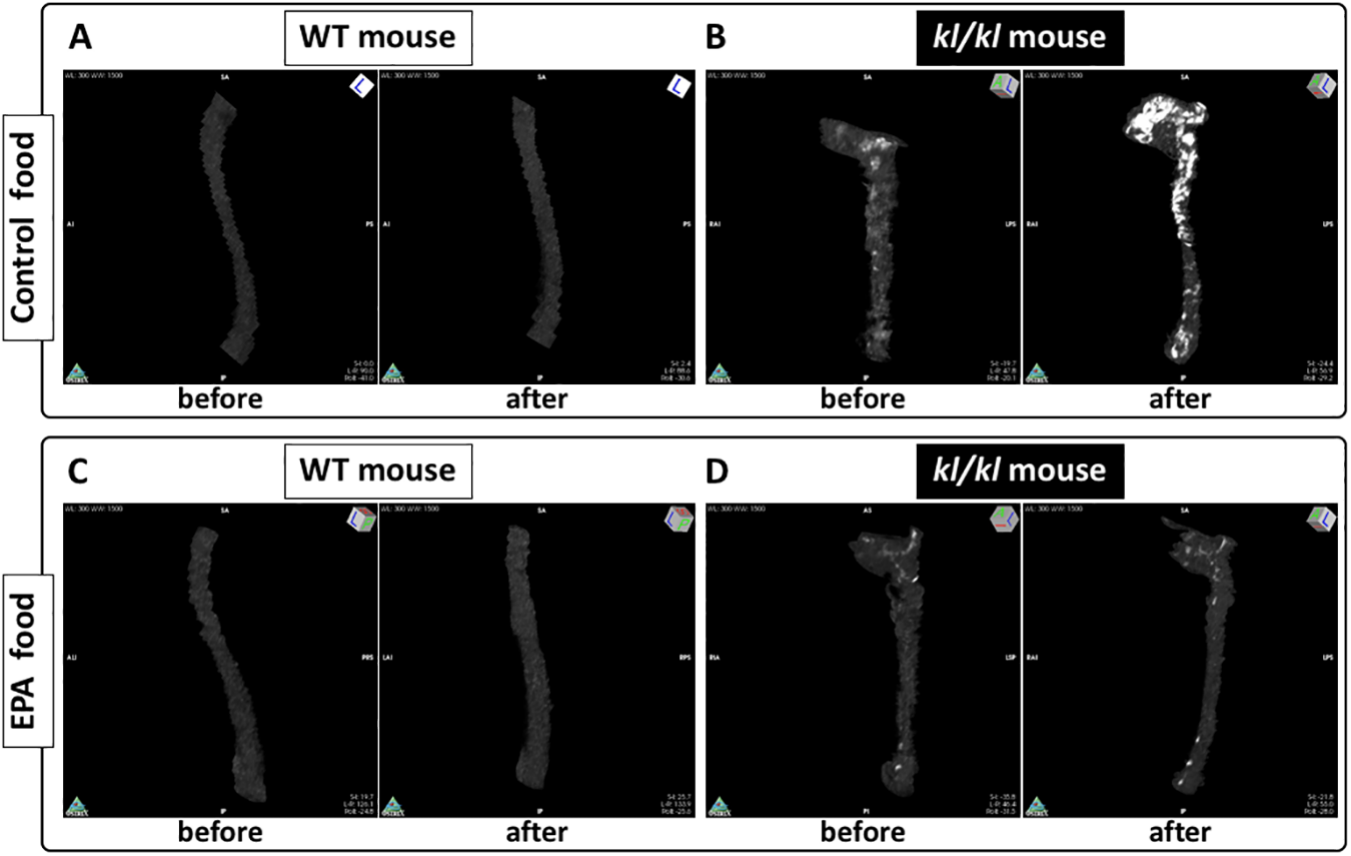
Representative CT images of thoracic and abdominal aortas in *klotho* mutant (*kl/kl)* and wild-type (WT) mice. A and B, a WT mouse (A) and a *kl/kl* mouse (B) before and after 4 weeks of control food (S1 and S2 Movies). C and D, a WT mouse (C) and a *kl/kl* mouse (D) before and after 4 weeks of EPA food (S3 and S4 Movies).

Calcium volume scores were significantly elevated in *kl/kl* mice after 4 weeks of control food (before vs after feeding, P < 0.05) (Fig 1B), but they were not elevated in *kl/kl* mice after 4 weeks of EPA food (before vs after feeding, P = NS) (Fig 1D). Calcium volume scores were not changed in WT mice after 4 weeks of control or EPA food (Fig 1A and 1C). The change in calcium volume score in control food-fed *kl/kl* mice (81±28 mm^3^) was significantly greater than that in control food-fed WT mice (8±6 mm^3^, P < 0.005) and that in EPA-fed *kl/kl* mice (18±17 mm^3^, P < 0.05) (Fig 1E). Fig 2 and S1 to S4 Movies show representative CT images of the thoracic and abdominal aortas in *kl/kl* and WT mice. Arterial calcification was elevated in klotho mice after 4 weeks of control food, but EPA prevented arterial calcification in *kl/kl* mice.

### Serum levels of EPA, AA, P, Ca and resolvin E1

Serum levels of EPA, arachidonic acid (AA), inorganic phosphorus (P) and calcium (Ca) (Table 1). Serum levels of EPA in EPA-fed *kl/kl* and WT mice were significantly increased compared to those in control-fed *kl/kl* and WT mice, and serum levels of AA in EPA-fed *kl/kl* and WT mice were significantly decreased compared to those in control-fed *kl/kl* and WT mice. The ratios of EPA to AA (EPA/AA) in EPA-fed *kl/kl* and WT mice were significantly larger than those in control-fed *kl/kl* and WT mice.

**Table 1:** Serum levels of EPA, AA, P and Ca.

		WT mice			*kl/kl* mice	
	Control food	EPA food	P value	Control food	EPA food	P value
**EPA (μg/mL)**	7.5 (18.1)	376.9 (142.0)	< 0.001	8.8 (7.9)	385.8 (169.8)	< 0.001
**AA (μg/mL)**	464.3 (224.5)	32.4 (11.53)	< 0.05	387.0 (191.2)	41.8 (20.0)	< 0.001
**EPA/AA**	0.01 (0.05)	11.13 (4.42)	< 0.01	0.02 (0.03)	8.08 (4.53)	< 0.001
**P (mg/dL)**	8.3±0.9	7.9±1.4	NS	16.4±1.5*	15.2±1.0#	NS
**Ca (mg/dL)**	8.9±0.2	8.9±0.2	NS	9.7±0.3	9.3±0.4	NS

WT: wild-type, *kl/kl*: *klotho* homozygous mutant, EPA: eicosapentaenoic acid, AA: arachidonic acid, P: phosphorus, Ca: calcium, NS: not significant.

*P<0.001: control food-fed WT vs *kl/kl* mice.

#P<0.001: EPA-fed WT vs *kl/kl* mice. Data are expressed as median (IQR) or mean ± SE.

Serum levels of P in control-fed klotho mice were increased compared to those in control-fed WT mice. EPA intake did not change the levels in klotho and WT mice. These results indicate that preventative effects of EPA on arterial calcification are not due to effects for negative phosphate balance.

Resolvin E1 is a lipid-derived mediator that is endogenously synthesized from EPA and is generated in response to inflammation and enhances the resolution phase of inflammation [21]. We measured serum levels of resolvin E1 in control food-fed and EPA food-fed WT mice. Serum levels of resolvin E1 in EPA-fed mice (n = 5) were significantly increased compared to those in control-fed mice (n = 6) (control: 1555±95 versus EPA: 1874±56 pg/mL, P = 0.01).

### Gene expression and activity of NOX

**Fig 3:**
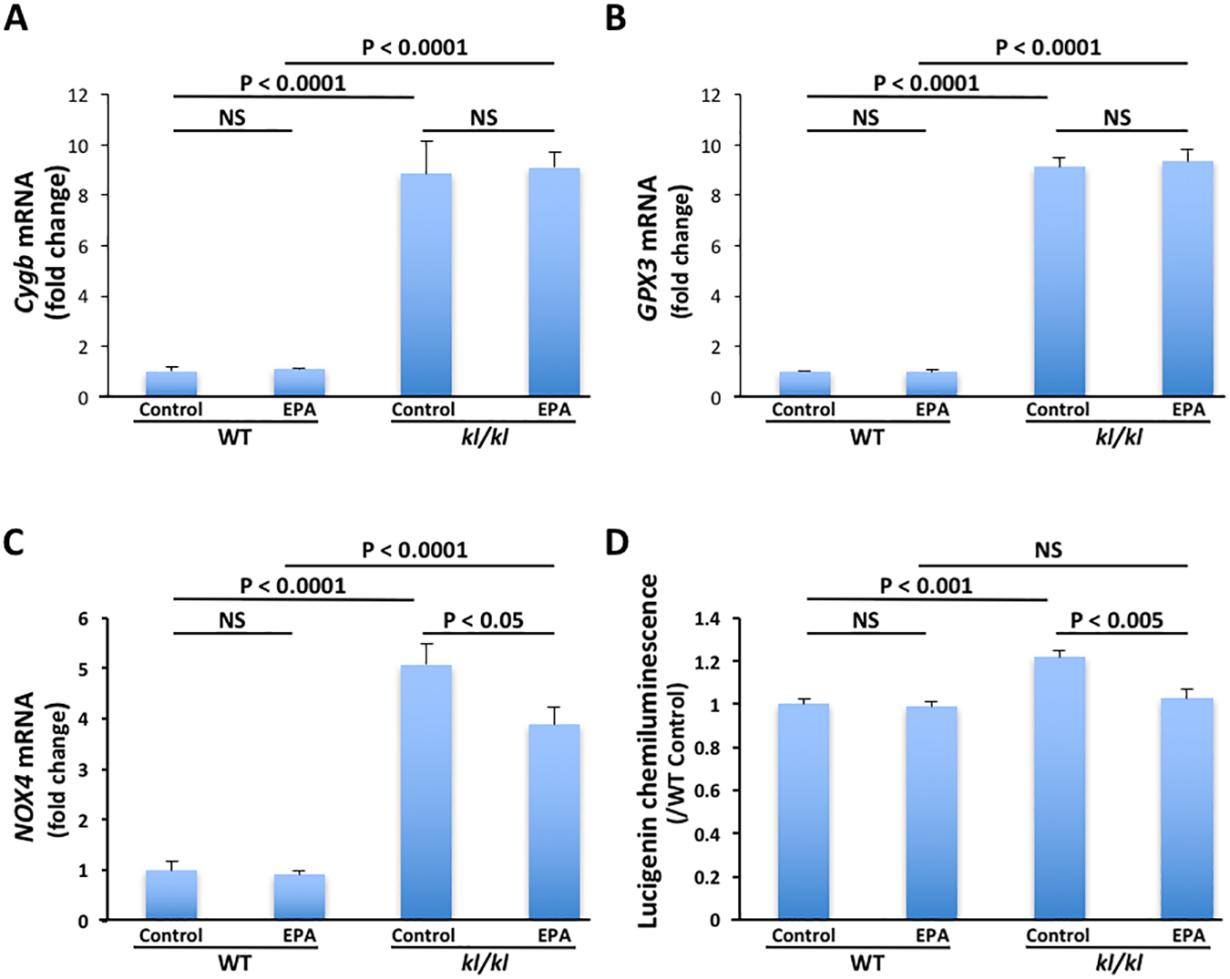
Gene expression of cytoglobin (*Cygb*), glutathione peroxidase 3 (*GPX3*) and NADPH oxidase (*NOX*) and activity of NOX. A to C, Expression levels of *Cygb* (A), *GPX3* (B) and *NOX4* (C) genes in arterial smooth muscle cells (SMCs) of wild-type (WT) and *klotho* mutant (*kl/kl)* mice treated with EPA and not treated with EPA (control) (n = 6, each). D, NOX activity in arterial SMCs of WT and *kl/kl* mice treated with EPA and not treated with EPA (control) (n = 6, each).

To further assess the mechanism of arterial calcification on *kl/kl* mice, we investigated the involvement of oxidative stress in arterial SMCs using the mouse oxidative stress and antioxidant defense PCR array of RT^2^ Profiler PCR Array. Expression levels of apolipoprotein E (*Apoe*), *NOX4*, uncoupling protein 2 (*Ucp2*), heat shock protein 1A (*Hspa1a*), flavin-containing monooxygenase 2 (*Fmo2*), cytoglobin (*Cygb*) and glutathione peroxidase 3 (*GPX3*) genes in arterial SMCs of a *kl/kl* mouse were upregulated compared to those in arterial SMCs of a WT mouse (Table 2). To investigate the involvement of the top 2 upregulated genes, we performed quantitative RT-PCR of *Cygb*, a globin molecule with a protective function during oxidative stress, and *GPX3*, an enzyme having anti-oxidative activity, in arterial SMCs treated with EPA and not treated with EPA. Expression levels of the *Cygb* and *GPX3* genes were significantly higher in arterial SMCs of *kl/kl* mice than in those of WT mice (Fig 3A and 3B). However, EPA did not decrease expression levels of the *Cygb* and *GPX3* genes.

**Table 2:** Genes on RT2 Prodiler PCR-array for which expression is up- or down-regulated in arterial SMCs of *kl/kl* mice.

Description	Symbol	Fold Regulation
Albumin	Alb	-3.398
Amyotrophic lateral sclerosis 2 (juvenile) homolog (human)	Als2	-1.007
Aldehyde oxidase 1	Aox1	-2.090
Adenomatosis polyposis coli	Apc	-1.048
Apolipoprotein E	Apoe	2.061
Ataxia telangiectasia and rad3 related	Atr	1.018
Catalase	Cat	-1.376
Chemokine (C-C motif) ligand 5	Ccl5	1.018
Copper chaperone for superoxide dismutase	Ccs	-1.181
Cathepsin B	Ctsb	1.121
Cytochrome b-245, alpha polypeptide	Cyba	-1.403
Cytoglobin	Cygb	16.246
Dynamin 2	Dnm2	1.282
Dual oxidase 1	Duox1	1.959
EH-domain containing 2	Ehd2	-1.089
Eosinophil peroxidase	Epx	-1.053
Excision repair cross-complementing rodent repair deficiency, complementation 2	Ercc2	1.045
Excision repair cross-complementing rodent repair deficiency, complementation 6	Ercc6	-1.154
Fanconi anemia, complementation group C	Fancc	-1.069
Flavin containing monooxygenase 2	Fmo2	3.828
Ferritin heavy chain 1	Fth1	1.034
Glutamate-cysteine ligase, catalytic subunit	Gclc	-2.853
Glutamate-cysteine ligase, modifier subunit	Gclm	1.125
Glutathione peroxidase 1	Gpx1	1.342
Glutathione peroxidase 2	Gpx2	-1.979
Glutathione peroxidase 3	Gpx3	16.279
Glutathione peroxidase 4	Gpx4	1.317
Glutathione peroxidase 5	Gpx5	-1.777
Glutathione peroxidase 6	Gpx6	-1.204
Glutathione peroxidase 7	Gpx7	1.989
Glutathione reductase	Gsr	1.039
Glutathione synthetase	Gss	1.006
Glutathione S-transferase kappa 1	Gstk1	-1.072
Glutathione S-transferase, pi 1	Gstp1	-1.066
Heme oxygenase (decycling) 1	Hmox1	-1.032
Heat shock protein 1A	Hspa1a	2.692
Isocitrate dehydrogenase 1 (NADP+), soluble	Idh1	1.600
Intraflagellar transport 172 homolog (Chlamydomonas)	Ift172	1.390
Interleukin 19	Il19	-1.147
Interleukin 22	Il22	-1.543
Keratin 1	Krt1	-1.058
Lactoperoxidase	Lpo	-1.640
Myoglobin	Mb	-3.055
Myeloperoxidase	Mpo	1.011
Neutrophil cytosolic factor 1	Ncf1	-2.478
Neutrophil cytosolic factor 2	Ncf2	-1.985
Neuroglobin	Ngb	-1.391
Nitric oxide synthase 2, inducible	Nos2	1.016
NADPH oxidase 1	Nox1	1.826
NADPH oxidase 4	Nox4	2.080
NADPH oxidase activator 1	Noxa1	1.127
NADPH oxidase organizer 1	Noxo1	-1.116
NAD(P)H dehydrogenase, quinone 1	Nqo1	1.624
Parkinson disease (autosomal recessive, early onset) 7	Park7	1.156
Peroxiredoxin 1	Prdx1	-1.205
Peroxiredoxin 2	Prdx2	-1.114
Peroxiredoxin 3	Prdx3	-1.035
Peroxiredoxin 4	Prdx4	1.067
Peroxiredoxin 5	Prdx5	-1.368
Peroxiredoxin 6	Prdx6	-1.494
Prion protein	Prnp	1.454
Proteasome (prosome, macropain) subunit, beta type 5	Psmb5	-1.160
Prostaglandin-endoperoxide synthase 1	Ptgs1	-1.029
Prostaglandin-endoperoxide synthase 2	Ptgs2	-2.986
Recombination activating gene 2	Rag2	-11.046
RecQ protein-like 4	Recql4	1.091
Stearoyl-Coenzyme A desaturase 1	Scd1	1.491
Serine (or cysteine) peptidase inhibitor, clade B, member 1b	Serpinb1b	1.938
Solute carrier family 38, member 1	Slc38a1	1.162
Superoxide dismutase 1, soluble	Sod1	-1.258
Superoxide dismutase 2, mitochondrial	Sod2	1.293
Superoxide dismutase 3, extracellular	Sod3	1.470
Sequestosome 1	Sqstm1	-1.400
Sulfiredoxin 1 homolog (S. cerevisiae)	Srxn1	-1.423
Thyroid peroxidase	Tpo	-1.223
Thioredoxin 1	Txn1	-1.687
Thioredoxin interacting protein	Txnip	-1.203
Thioredoxin reductase 1	Txnrd1	-1.399
Thioredoxin reductase 2	Txnrd2	-1.518
Thioredoxin reductase 3	Txnrd3	1.316
Uncoupling protein 2 (mitochondrial, proton carrier)	Ucp2	2.547
Uncoupling protein 3 (mitochondrial, proton carrier)	Ucp3	-1.173
Vimentin	Vim	1.370
Xeroderma pigmentosum, complementation group A	Xpa	1.372

NOX is an enzyme that produces superoxide and plays a pivotal role in generation of oxidative stress in vascular SMCs [22]. Other upregulated genes in PCR array are related to proteins that have antioxidant properties. To clarify the involvement of NOX, we performed quantitative RT-PCR of *NOX4*, a component of NOX, and measured NOX activity in arterial SMCs treated with EPA and not treated with EPA. Expression level of the *NOX4* gene and activity of NOX were significantly higher in arterial SMCs of *kl/kl* mice than in those of WT mice (Fig 3C and 3D). EPA decreased expression levels of the *NOX4* gene and NOX activity.

### GPR120 and NOX

**Fig 4:**
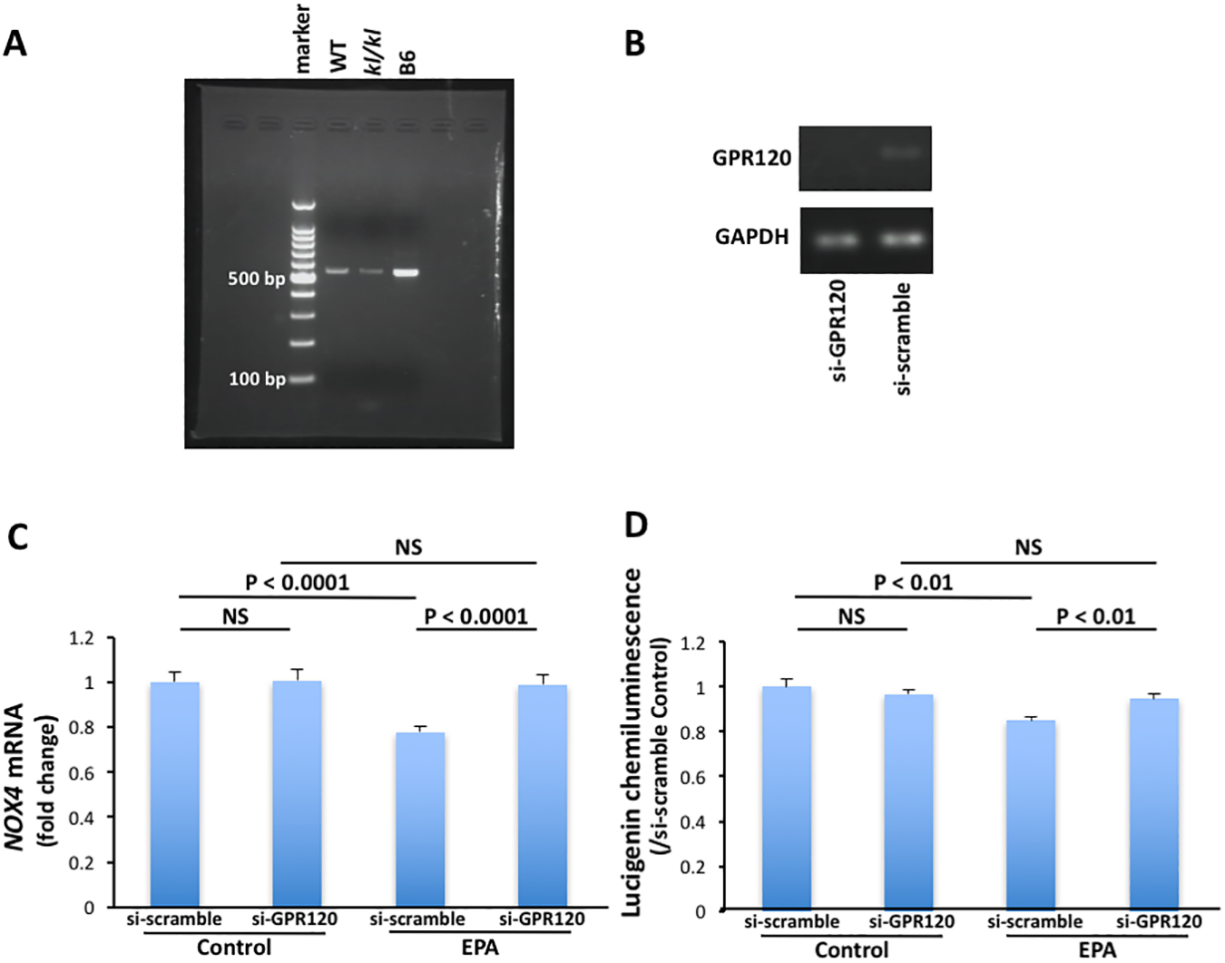
GPR120 and NADPH oxidase (NOX) in arterial smooth muscle cells. A, Expression of the GPR120 gene. Lane 1, maker; lane 2, wild-type (WT) mouse; lane 3, *klotho* mutant (*kl/kl)* mouse and lane 4, C57/BL6 mouse (positive control). B, GPR120 downregulation by siRNA. Lane 1, si-GPR120 RNA and lane 2, s-scramble RNA. C, Effects of GPR120 gene knockdown on *NOX4* gene expression and NOX activity in arterial SMCs of *kl/kl* mice (n = 6, each).

G-protein-coupled Receptor 120 (GPR120) is a receptor for n-3 fatty acids [20]. Activation of GPR120 by n-3 fatty acids inhibits inflammation cascades and preserves insulin sensitivity. We hypothesized that inhibitory effects of EPA on NOX expression and activity might be actions on arterial SMCs via GPR120. mRNA of the gene encoding GPR120 was expressed in arterial SMCs obtained from *kl/kl* and WT mice (Fig 4A).

Next, we investigated effects of *GPR120* gene knockdown on *NOX4* gene expression and NOX activity in arterial SMCs of *kl/kl* mice. GPR120 downregulation by siRNA canceled the effects of EPA on *NOX4* gene expression and NOX activity in arterial SMCs of *kl/kl* mice (Fig 4B, 4C and 4D).

## Discussion

Two major findings were obtained in the present study. First, EPA intake prevented arterial calcification in *klotho* mutant mice. Second, *NOX* gene expression and activity were elevated in arterial SMCs of *klotho* mutant mice and EPA reduced them via GPR120.

Elevated oxidative stress is associated with the development of vascular calcification [11, 12]. Oxidative stress induces vascular SMC calcification by upregulation of Runx2, which is mediated by activation of the AKT/FOXO1/3 signaling axis. Although there are several enzymatic sources of reactive oxygen species (ROS) in vascular cells, NOX is thought to be a major enzymatic source of ROS in atherosclerosis [23]. Gao et al reported that *klotho* deficiency upregulated NADPH oxidase activity and superoxide production in the media of aortas[24]. We also showed that NOX gene expression and activity were elevated in arterial SMCs of *klotho* mutant mice. Although the precise mechanism underlying the inhibitory effects of EPA on vascular calcification remains unclear, reduction of NOX activity by EPA might play an important role in prevention of vascular calcification in *klotho* mutant mice.

Changes of NOX4 gene expression and NOX activity were minor in our study using a cell culture system. Another mechanism might also contribute to the prevention of vascular calcification *in vivo*. Serum levels of resolvin E1, a bioactive metabolite of EPA, were increased in EPA food-fed mice. Resolvin E1 is responsible for facilitating the resolving phase of acute inflammation [25]. Furthermore, resolvin E1 reduces oxidative stress by suppressing NOX activation [26]. These effects might contribute to the prevention of vascular calcification. Further studies are needed to clarify this point.

Coronary artery calcium assessed by multi-detector CT reflects the plaque burden of coronary arteries, and it is an independent predictor of cardiovascular events and is associated with the incidence of congestive heart failure [2, 27]. Furthermore, one of the most appealing features of CT is the potential to detect the progression or regression of coronary atherosclerotic disease noninvasively [28]. CT also enables evaluation of vascular calcification in living mice [29]. We assessed changes in the degree of arterial calcification by multi-detector CT as recent clinical situations in this study. Our study showed that CT is useful for detecting the changes over time and by treatment in coronary calcification of living mice.

In the Japan EPA lipid intervention study (JELIS) in patients with hypercholesterolemia, on-treatment mean plasma EPA concentrations and EPA/AA were 170 μg/mL and 1.21, respectively in patients treated with EPA (1800 mg/day) [30]. Therefore, serum levels of EPA in EPA-fed mice were very high in this study (EPA-fed WT mice, median (IQR): 376.9 (142.0), mean ± SD: 373.4 ± 75.4 μg/mL; EPA-fed *kl/kl* mice, median (IQR): 385.8 (169.8), mean ± SD: 393.5 ± 150.1 μg/mL), and EPA/AA ratios in EPA-fed mice were also quite high (EPA-fed WT mice, median (IQR): 11.13 (4.42), mean ± SD: 11.57 ± 2.39; EPA-fed *kl/kl* mice, median (IQR): 8.08 (4.53), mean ± SD: 9.15 ± 3.32). A large amount of EPA might be needed for clinical application of this study. Since it was found in this study that EPA reduced NOX activity via GPR120, novel GPR120 agonists might also be useful for prevention of arterial calcification.

A decrease in the expression level of klotho is associated with arterial calcification and stiffness in patients with CKD [5–7]. Thus, preventative effects of EPA on atrial calcification might be expected in patients with CKD.

In conclusion, EPA intake prevents arterial calcification along with reduction of NOX gene expression and activity via GPR120 in *klotho* mutant mice.

## Supporting information

S1 Movie CT images of thoracic and abdominal aortas in a *klotho* mutant *(kl/kl)* mouse before control food. (MOV)

S2 Movie CT images of thoracic and abdominal aortas in a *klotho* mutant *(kl/kl)* mouse after 4 weeks of control food. (MOV)

S3 Movie CT images of thoracic and abdominal aortas in a *klotho* mutant *(kl/kl)* mouse before EPA food. (MOV)

S4 Movie CT images of thoracic and abdominal aortas in a *klotho* mutant *(kl/kl)* mouse after 4 weeks of EPA food. (MOV)
